# A Comparative Study of Surfactant Solutions Used for Enhanced Oil Recovery in Shale and Tight Formations: Experimental Evaluation and Numerical Analysis

**DOI:** 10.3390/molecules29143293

**Published:** 2024-07-12

**Authors:** Weidong Chen, Xiangfei Geng, Bin Ding, Weidong Liu, Ke Jiang, Qinglong Xu, Baoshan Guan, Lin Peng, Huan Peng

**Affiliations:** 1 Key Laboratory of Oilfield Chemicals, China National Petroleum Corporation, Beijing 100083, China; gengxf@petrochina.com.cn (X.G.); lwd69@petrochina.com.cn (W.L.); pengl21@pewtrochina.com.cn (L.P.);; 2 PetroChina Research Institute of Petroleum Exploration & Development, Beijing 100083, China; 3 National Key Laboratory of Continental Shale Oil, PetroChina Daqing Oilfield Research Institute of Petroleum Exploration & Development, Daqing 163000, China

**Keywords:** surfactant solutions, enhanced oil recovery, shale and tight reservoir, evaluation and modeling

## Abstract

Applying chemical enhanced oil recovery (EOR) to shale and tight formations is expected to accelerate China’s Shale Revolution as it did in conventional reservoirs. However, its screening and modeling are more complex. EOR operations are faced with choices of chemicals including traditional surfactant solutions, surfactant solutions in the form of micro-emulsions (nano-emulsions), and nano-fluids, which have similar effects to surfactant solutions. This study presents a systematic comparative analysis composed of laboratory screening and numerical modeling. It was conducted on three scales: tests of chemical morphology and properties, analysis of micro-oil-displacing performance, and simulation of macro-oil-increasing effect. The results showed that although all surfactant solutions had the effects of reducing interfacial tension, altering wettability, and enhancing imbibition, the nano-emulsion with the lowest hydrodynamic radius is the optimal selection. This is attributed to the fact that the properties of the nano-emulsion match well with the characteristics of these shale and tight reservoirs. The nano-emulsion is capable of integrating into the tight matrix, interacting with the oil and rock, and supplying the energy for oil to flow out. This study provides a comprehensive understanding of the role that surfactant solutions could play in the EOR of unconventional reservoirs.

## 1. Introduction

China is undergoing a “Shale Revolution”. It is essential to consider the role that chemistry can play in improving oil recovery from oil and tight reservoirs.

### 1.1. How Does Chemicals’ EOR Start in Conventional Reservoirs?

Chemical agents have been widely used for a long time in petroleum exploration and development. Considerable quantities of chemicals are added to drilling fluids, completion fluids, fracturing fluids, and fluids used in EOR operations [1,2,3]. Traditional chemicals include inorganic salts, organic chemicals, a solvent, a surfactant, and water/oil-soluble polymers [4]. Aqueous systems containing these chemicals are utilized to stimulate oil and gas production. In sandstone and carbonate reservoirs, alkali–surfactant–polymer (ASP) flooding and surfactant polymer (SP) flooding have aided in tens of millions of tons of oil production [5,6,7,8]. The mechanism of ASP flooding is to improve the mobility ratio and increase the capillary number, mainly by reducing the oil–water interfacial tension (IFT). ASP is used in cases involving more than 20% OOIP and has been successful in projects in North America as well as in China, especially in the Daqing Oilfield [9,10].

As the focus of industry and research shifts towards unconventional reservoirs, chemical EOR may play a greater role and should receive more attention.

### 1.2. Why Chemical EOR Is Needed in Tight and Shale Oil?

The development of shale has achieved great success in North America. At present, China is pursuing its own Shale Revolution but is faced with much greater challenges. The relatively low maturity of the continental shale oil leads to the problem of low oil fluidity, in addition to the ultra-low permeability caused by the tight matrix [11,12]. The pores and throats of shale rocks in China have nanoscale sizes and are unconnected. Compared to shale from Eagle Ford or Bakken, it is much more difficult to re-saturate China’s shale with oil. Similarly, it is almost impossible to drive flooding water into the shale.

Hydraulic fracturing is now the main strategy for developing shale. However, the formation energy supply provided by fracturing is temporary, and daily production decreases rapidly [13]. Efficient aqueous surfactant solutions need to be injected into the reservoir to “re-energize” the formation and achieve incremental oil recovery. This is a great challenge both for petroleum and chemical engineers. The key technical issues are how to inject fluids into the tight matrix and produce oil from the micro- and nanopores [14].

### 1.3. What Kinds of Solutions Are Used in Chemical EOR?

With the progress of chemical engineering and nano-technology, recent research has proposed a few aqueous surfactant solutions that are promising in the EOR of shale and tight formations [15,16,17,18,19], as summarized in Table 1. Limited by the pore size of a shale matrix, polymers are unfavorable and surfactants are the dominant agent.

Traditional surfactant solutions including anionic, cationic, non-ionic, zwitteric, and gemini surfactants are used in EOR projects. Micro-emulsions (MEs) are defined as transparent, optically isotropic, thermodynamically stable, and monophasic dispersions composed of water, oils, surfactants, and cosurfactants. Micro-emulsions are very promising in enhanced oil recovery [20]. Micro-emulsion techniques in EOR can be divided into two types. In the first type, the oil reservoir is flooded with water containing a small percentage of surfactants and other additives. This surfactant solution reacts with the reservoir’s trapped oil and creates a micro-emulsion underground. In the second type, the micro-emulsion is formed on the ground by mixing a surfactant with hydrocarbon components in the water, and then the micro-emulsion slug is injected into the reservoir. Both the surfactant and hydrocarbon in the second type of micro-emulsion could react with the trapped oil. In this study, when we talk about micro-emulsion, we mean the second type. Micro-emulsions are also referred to as nano-emulsions in some studies since the droplet size is in the order of 100 nm.

Nano-fluids composed of nano-sized particles, surfactants, or micro-emulsion are gradually being applied in the shale and tight EOR field. For example, in a study, an aqueous solution of anionic surface-active agents containing light non-ferrous-metal nanoparticles was used in EOR [21]. The mechanism of nano-fluids is unclear. The common understanding is that nano-fluids can reduce oil/water interfacial tension, alter wettability, and induce structural disjoining pressure. Aside from the structural disjoining pressure mechanism, nano-fluids actually have similar effects to surfactant solutions. Thus, nano-fluids are discussed together with surfactants and micro-emulsion in this study.

### 1.4. Why a Comparison Analysis Is Necessary

All the EOR projects are faced with the problem of incremental cost of chemical agents. The cost increase caused by the use of chemicals is undoubtedly a burden on oil companies and must be compensated by increasing oil production. Therefore, a comparison study on chemicals is indispensable as it can guide the selection of the best aqueous system under limited cost constraints.

The traditional understanding of chemical EOR is that the chemicals in the aqueous phase reduce the oil–water interfacial tension and change the liquid–rock contact angle, thus promoting the imbibition of water into the rock matrix. The calculation of capillary pressure by the Young–Laplace equation (Equation (1)) is the core of this understanding. In high- and medium-permeability reservoirs (K > 10 md) and some low-permeability reservoirs (K > 1 md), the effect of additive chemical is to eliminate the resistance caused by capillary pressure. When interfacial tension is ultra-low (at the dimension of 10^−3^ mN/m), water flooding efficacy in the reservoir is improved. In shale and tight reservoirs, the major recovery driving mechanisms are still under discussion [22]. It was generally believed that by altering the wettability to water-wet, the capillary pressure could drive the aqueous phase into the pore and expel the crude oil out. This is just a simplified and rough description.

(1) $$P_{c}=\frac{2\sigma \;cos\theta }{r}$$

$P_{c}$ = capillary pressure,
θ = contact angle of liquid on the rock surface,
σ = interfacial tension of water with oil, and
r = radius of the porous media.

However, the size effects of nanoscale pores and the throat system are not well considered. For example, two chemicals with a hydrodynamic radius of 100 nm and 10 nm, respectively, can produce similar IFT and contact angle results by adjusting the concentration and salinity. But the difficulty of these two chemical systems entering the 50 nm pore is completely different. The capillary equation cannot describe the resistance caused by this size effect. In addition, the size of oil droplets also matters during their flow process. It is speculated that small oil droplets flow more easily and quickly than large oil ones. In summary, it is proposed that attention should also be paid to the ability of chemical substances to enter nanometer pores and increase the fluidity of oil, in addition to reducing interfacial tension and altering wettability.

### 1.5. What Is the Research Novelty of This Comparison Study?

This study proposes a series of evaluations and modeling of different aqueous solutions used in the chemical EOR of shale and tight reservoirs. Through comparative study at three scales, the performance of surfactant solutions is comprehensively and systematically demonstrated from their morphology and basic properties, microscopic oil-displacing performance, and macroscopic oil-increasing effect. Researchers can fully understand the methods for screening chemicals. Identification characteristics of chemicals can be obtained from this study.

As we discussed previously, the micro–nanopore and throat systems are the foundation for the ultra-low permeability and low connectivity in tight and shale reservoirs. This study addresses the significance of droplet size of surfactant solutions and their ability to enter the nanopores of the tight matrix. This is the most critical property of aqueous solutions, in addition to altering IFT and wettability as basic mechanism.

A heterogeneous microfluidic device has been developed to demonstrate the properties of chemicals in improving the fluidity of the crude oil. Numerical simulation of the chemical re-energizing the reservoir is conducted to model the behavior of the aqueous chemical system in the field scale. Although numerical models are mostly based on convention mechanisms including IFT and wettability, we attempt to combine them with threshold pressure gradients, which describe the difficulty of the solution entry into the matrix. Using this model, this study shows how and where surfactant solutions “modify” reservoirs. Due to the inability of traditional oil recovery models to be directly applied, it is very difficult to model the chemical EOR operation in unconventional reservoirs. This study is a preliminary exploration of modeling the chemical EOR after fracturing. Further studies are needed in order to better simulate the impact process of surfactant solution on reservoirs.

This study provides a through understanding on how chemicals can enhance shale oil recovery from nanoscale to mesoscale and then to the oilfield scale. It has guiding significance for chemical EOR projects which intend to boost the shale and tight oil production and realize an industrial revolution.

## 2. Materials and Methods

Four types of aqueous solutions are used for performance evaluation in this comparison study. They include betaine-type surfactant (TCJ), micro-emulsion imbibition enhancement agent (MEI), micro-emulsion nanomaterial (MEN), and solid particle nanomaterial (SPN). MEI and MEN are two micro-emulsions with different properties. These systems represent commonly used aqueous solutions in the EOR of unconventional reservoirs. In order to better understand their performance differences and respective application prospects, comparison and evaluation of the chemicals were divided into three parts.

### 2.1. Tests of Chemical Morphology and Properties

This section of testing includes interfacial tension and wettability measurement, hydrodynamic radius (particle size) measurement, micro-morphology observation of chemicals applying TEM, and evaluation of the adsorption rate and core damage. The evaluation of morphology and properties is the key to the role of chemicals in shale and tight formations. Only chemicals that meet these standards are candidates for unconventional reservoir stimulation agents. For example, chemicals with high Molecular Weight (>10,000) have a large particle radius and are unlikely to penetrate into the tight matrix. Similarly, only chemicals with high interfacial activity can improve the imbibition of aqueous phase into shale.

#### 2.1.1. Interfacial Tension and Wettability Measurement

The IFT of crude oil with chemical solutions was measured by the spinning drop method, which is suitable for measuring low IFTs ranging from 1 mN/m to 10^−5^ mN/m [23]. The crude oil was retrieved from Jilin Basin, Xinjiang Oilfield, Sichuan Basin, and Ordos Basin. The IFT of the liquid paraffin with aqueous phase was also measured as a reference.

The wettability-altering capability of the aqueous chemical solution was evaluated by measuring the contact angles between the solution and the surfaces of water-wet SiO_2_, oil-wet SiO_2_, and rock chips. The contact angles were measured by the sessile drop method. Usually, a lower contact angle indicates lower frictional force during the flow process of a solution on a solid surface.

#### 2.1.2. Hydrodynamic Radius Measurement and Micro-Morphology Observation

The hydrodynamic radius (Rh) of the aqueous solutions and the distribution of Rh were measured by the dynamic light scattering (DLS) technique. The wavelength was set as 532 nm and scattering light angle was 90 degree. The DLS method assumes that all the chemical particles are spherical in nature [24]. It only requires a small amount of materials, has a relatively low cost, high production capacity, and is easy to carry out [25].

The micro/nano-morphology of the aqueous solutions was studied using transmission electron microscopy (TEM). Photos of the prepared nano-sample were taken, in which the micro-structure and size of the particles could be observed. TEM has extremely high resolution and is currently the highest resolution method in microscopic imaging, with an actual resolution of approximately 0.2 nm. TEM can help us understand the microscopic morphology information of aqueous systems.

#### 2.1.3. The Adsorption Rate and Core Damage Assessment

Four aqueous solutions were prepared into critical micelle concentration (CMC) solution with synthetic brine. Weigh the corresponding oil sands and chemical solutions, respectively, and oscillate for 24 h at the formation temperature each time. Centrifuge and measure surface tension using an upper layer of clarified liquid. The adsorption loss rate of a solution is calculated by the change in surface tension.

High damage to the formation caused by chemical systems is not desired [26]. The core damage rate is determined based on the change in permeability of low-permeability (K~30 md) cores. The core was first saturated with experimental water under pressure, and the initial permeability of the core was measured. The core was then saturated with oil until the sample reached a bound water state. Drive oil-saturated rocks through aqueous solutions and measure the permeability of the core after displacement. The core damage rate is calculated by comparing the changes in permeability before and after displacement.

### 2.2. Analysis of Micro-Oil-Displacing Performance

This section of testing includes threshold pressure gradient (TPG) determination, microfluidic modeling testing, and core injection combined with low-field nuclear magnetic resonance (NMR). The flow of aqueous systems in nanopores cannot be directly visualized. Thus, a physical model is designed to simulate the flow of chemical solutions in the pore throats of shale and tight reservoirs. Although they are pseudo visualizations of the nanoscale world, they can reveal the mechanisms by which the aqueous systems displace oil.

#### 2.2.1. Threshold Pressure Gradient Determination

It is believed that a minimum pressure gradient is required before the liquid begins to flow in a porous medium [27], which is commonly referred to as the threshold pressure gradient or starting pressure gradient. In shale and tight formations, the more difficult the fluid flow is, the greater the threshold pressure gradient. Usually, the TPG is determined through numerical equation calculations or through core injection. The disadvantage of core injection is its pore repeatability and high error.

**Figure 1:**
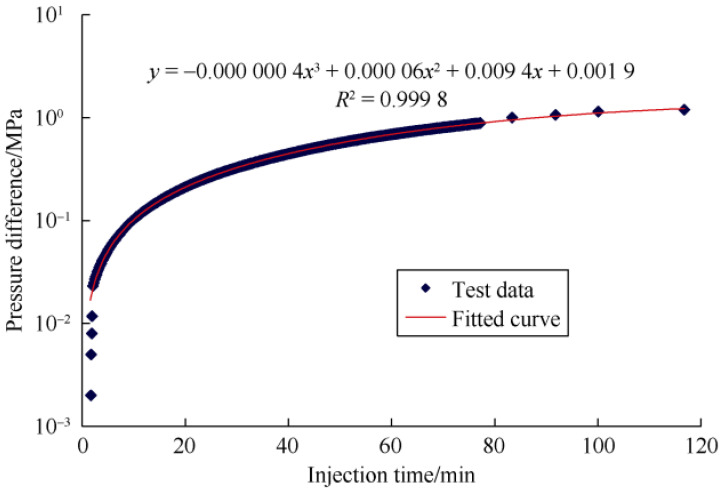
Determination of the critical threshold pressure by fitting the curve of injection time vs. Pressure difference. Reproduced with permission from Ref. [12] Copyright 2020 Elsevier.

A capillarity analysis system was used in this study to determined the threshold pressure gradient. Different aqueous solutions were injected at certain speed (0.1 mL/min) into the hydrophilic and hydrophobic capillaries with the length of 60 cm and inner diameter of 1 μm or 0.3 μm. As shown in Figure 1, the pressure difference is plotted against the injection time and the curve is fitted. A critical threshold pressure is determined by extrapolating to zero. The TPG is calculated by dividing the critical pressure by the length of the capillary tube.

#### 2.2.2. Micro-Fluidic Modeling

Microscopic glass models with pore throat structures are widely used to model fluid flow in tight matrix. In this study, a microfluidic model with a pore depth of 15 μm, a throat depth of 2 μm and a pore volume of 10 mm^3^ were used to observe the oil flow enhancement effect of the chemical system. One drawback of this model is that the pores are all the same, making it a homogeneous model.

**Figure 2:**
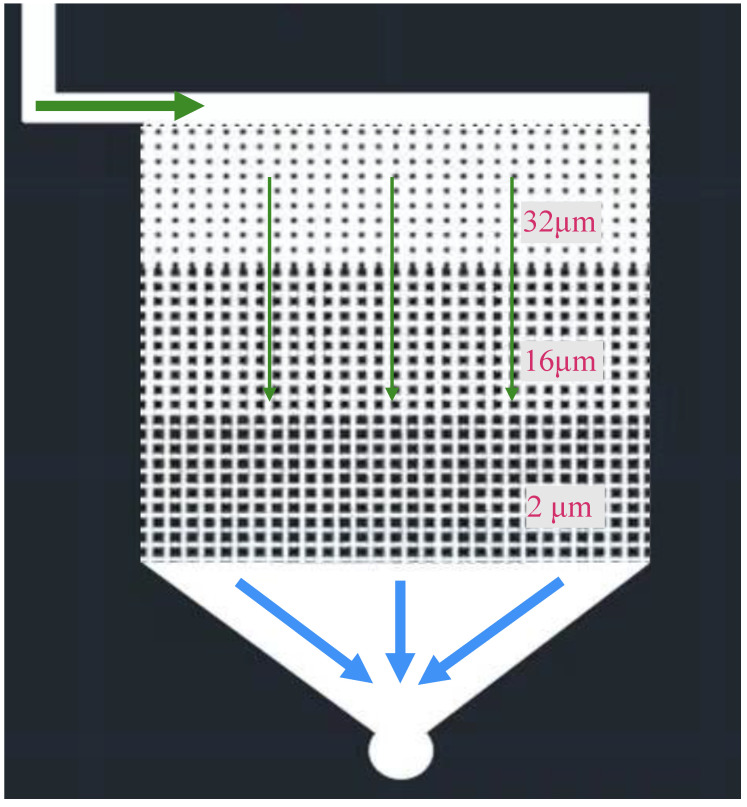
Heterogeneous micro-fluidic model simulating different pores of the rock matrix.

To compensate for this deficiency, this study applied a heterogeneous model to observe the small-size oil effect in different pore throat systems. Figure 2 shows the structure of the model, where the throats were etched at 32 μm, 16 μm, and 2 μm, respectively. The model was saturated with shale oil, and then the aqueous systems was injected into the model at certain speed. The fluid first flowed into the high-permeability zone (32 μm), then into the medium-permeability zone (16 μm), and the low-permeability zone (2 μm). The shape and flow behavior of oil droplets were observed and recorded using a high-speed digital camera.

#### 2.2.3. Core Injection with Low-Field NMR

Low-field NMR was used to monitor the amount of fluid injected into the pores of the rock core. The core injection testing device includes a low-field nuclear magnetic analyzer and a high-temperature and high-pressure displacement device. The T2 spectrum (transverse relaxation time) measured by low-field NMR technology is related to the pore size of the rock [28]. The value of T2 is positively correlated with the pore radius. By measuring the T2 of the cores saturated with the crude oil or injected with aqueous phase, penetration of liquids into the nanopores can be monitored.

This method can be used to compare the injection capacity of different fluids in tight matrix. In shale and tight formations, only an aqueous system that can flow into the tiny pores can achieve true oil displacement effects. Therefore, the high injection or penetration capacity of injected fluids is an ideal choice for unconventional EOR.

### 2.3. Simulation of Macro-Oil-Increasing Effect

There is a certain gap between the laboratory experimental results and the on-site pilot test results. When it comes to simulating macroscopic oil displacement effect of chemical fluids in oilfields, laboratory methods are limited. The evaluation and prediction of the oil-increasing effect of chemicals at the oilfield scale rely on numerical simulation. In this comparison study, a fracture geological model was established, and on this basis, aqueous solutions were injected and a huff-n-puff process was conducted. The sweeping area and oil-increasing effects of water, surfactant, and micro-emulsion were evaluated. This also helps to understand the mechanism by which chemicals enhance oil recovery in shale and tight formations.

#### 2.3.1. Fractured Geological Model

**Figure 3:**
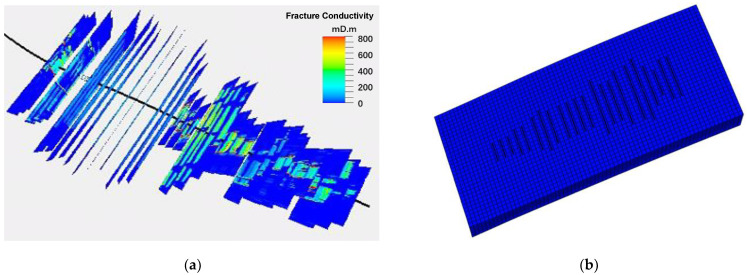
Fracture model established based on the Jimusaer geological setting and fitted by the pumping pressure curve. (**a**) Integrated simulation of fractures; (**b**) Characterizing fractures.

Geological modeling is based on learning from geophysical data, well logging data, seismic data, etc. [29]. The fracture model is established adapting from the geological model of Jimusaer shale formation in Junggar Basin, Xinjiang Oilfield. The fracture propagation and parameters were determined through history matching of pumping pressure curves [30]. A comprehensive sweet spot (CSS) method was used in the establishment of fracture model, dividing the sweet spot into the geological sweet spot (GSS) and the engineering sweet spot (ESS) [31,32]. Figure 3 shows the fracture model working as the initial model before injecting the aqueous solution.

#### 2.3.2. Mechanisms of Chemicals EOR

The aqueous solutions improve oil recovery from formations mainly through reducing oil–water IFT, altering wettability, and enhancing oil solubility and fluidity. In the numerical model, the IFT reduction is related by the capillary number, and wettability alteration effect is a function of adsorption. In the case of micro-emulsion, the fraction of surfactant in the emulsion phase also affect the IFT value. In the model, the relative permeability curve is generated by Equations (2)–(6) [33,34].

(2) $$IFT=\frac{C}{{\left(\frac{V_{x}}{V_{s}}\right)}^{2}}$$

(3) $$N_{c}=\frac{\mu v}{IFT}$$

(4) $$K_{rw,ow}^{IFT}=(1-\gamma )K_{rw,ow}^{low}+\gamma K_{rw,ow}^{high}$$

(5) $$K_{ro,ow}^{IFT}=(1-\gamma )K_{ro,ow}^{low}+\gamma K_{ro,ow}^{high}$$

(6) $$\gamma =\frac{\left.log(N_{c})-log(N_{c}^{low}\right)}{\left.log(N_{c}^{high})-log(N_{c}^{low}\right)}$$

In Equation (2), *IFT* is the interfacial tension of the system with oil, V_s_ is the volume of surfactant used, *V_x_* is the volume of the micro-emulsion phase, C is a constant derived from fitting the *IFT* against (*V_x_/V_s_*)^2^. In Equation (3), N_c_ is the capillary number, μ is the displacing fluid (water) viscosity, and υ is the interstitial velocity of water. In Equations (4) and (5), $K_{rw,ow}^{IFT}$($K_{ro,ow}^{IFT}$) is the relative permeability to water (oil) of the oil-wet rock after IFT reduction effect, $K_{rw,ow}^{low}$ ($K_{rw,ow}^{low}$) and $K_{rw,ow}^{high}$ ($K_{ro,ow}^{high}$) are the relative permeability to water (oil) of the oil-wet rock at low and high interfacial cases. γ is the interpolation parameter for the IFT reduction.

The wettability alteration is modeled through adsorption phenomenon, using the Langmuir adsorption model. It is given by Equations (7)–(11).

(7) $$Ads=\frac{tad1*\chi_{surf}^{W}}{1+tad2*\chi_{surf}^{W}}$$

(8) $$\left.K_{ro}=K_{ro,ow}^{IFT}+\omega (K_{ro,ww}-K_{ro,ow}^{IFT}\right)$$

(9) $$\left.K_{rw}=K_{rw,ow}^{IFT}+\omega (K_{rw,ww}-K_{rw,ow}^{IFT}\right)$$

(10) $$\left.P_{c}=P_{c,ow}^{IFT}+\omega (P_{c,ww}-P_{c,ow}^{IFT}\right)$$

(11) $$\omega ={\left(\frac{\frac{1}{Ads-Ads^{l}+\varepsilon_{y}}-\frac{1}{\varepsilon_{y}}}{\frac{1}{Ads^{h}-Ads^{l}+\varepsilon_{y}}-\frac{1}{\varepsilon_{y}}}\right)}^{n_{y}}$$

In Equation (7), ads is the mole fraction of chemicals in the adsorbed phase, $\chi_{surf}^{W}$ is the mole fraction in the liquid phase, tad1 is the first parameter in the Langmuir isotherm, and tad2 is the second parameter in the Langmuir isotherm. Equations (8)–(11) are similar to Equations (4)–(6). In Equation (11), ω is the interpolation parameter for the wettability alteration, $Ads^{l}$ ($Ads^{h}$) is the mole fraction of effective component in the adsorbed phase in completely water-wet (oil-wat) rock, $\varepsilon_{y}$ is the first weighting factor, and $n_{y}$ is the second weighting factor which will create a tendency behavior within the interpolation.

The oil solubility enhancement effect is modeled by equilibrium constant, which is given by Equations (12)–(14).

(12) $$K_{oil}^{om}=\frac{\chi_{oil}^{m}}{\chi_{oil}^{o}}$$

(13) $$K_{water}^{om}=\frac{\chi_{water}^{o}}{\chi_{water}^{m}}$$

(14) $$K_{surf}^{om}=\frac{\chi_{surf}^{o}}{\chi_{surf}^{m}}$$

where $\chi_{oil}^{m}$ is the mole fraction of oil components in the micro-emulsion phase, $\chi_{oil}^{o}$ is the mole fraction of oil components in the oil phase, $\chi_{water}^{o}$ is the mole fraction of water in the oil phase, $\chi_{water}^{m}$ is the mole fraction of water in the micro-emulsion phase, $\chi_{surf}^{o}$ the mole fraction of surfactant in the oil phase, and $\chi_{surf}^{m}$ is the mole fraction of surfactant in the micro-emulsion phase.

#### 2.3.3. The Threshold Pressure Gradient in Simulation

**Figure 4:**
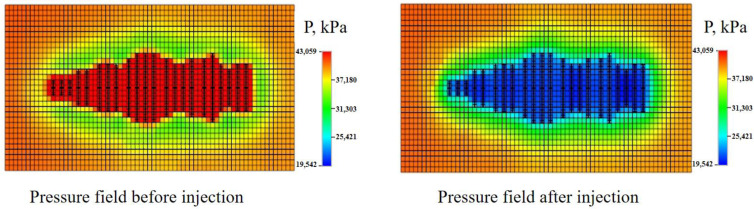
The pressure field of the model before and after the chemical injection and huff-n-puff process.

The threshold pressure gradient determined from lab experiment was applied in the simulation to consider the pressure field of different chemicals. A difference method is used comparing the pressure before and after the huff-n-puff process. The pressure effect range is given by the following equation.

(15) $$S_{p}=P_{f}^{after}-P_{f}^{before}-\Delta P$$

where *S*_*p*_ is the area affected by the pressure change due to injection of aqueous systems, $P_{f}^{after}$ if the pressure field after huff-n-puff process, $P_{f}^{before}$ is the pressure field before huff-n-puff process, and ∆P is the threshold pressure gradient. The change in pressure field due to the injection is illustrated in Figure 4. A low-threshold pressure gradient means that the injection of chemical can affect a wider area in the panel.

## 3. Results and Discussions

Through a series of experiments and their result, this study describes and explains the differences of the commonly used chemical systems in the EOR process of shale and tight reservoirs. The application prospects of these chemicals were analyzed and discussed on the basis of experimental and simulation results.

### 3.1. Chemical Morphology and Properties

#### 3.1.1. IFT Reduction and Wettability Alteration Effects

**Figure 5:**
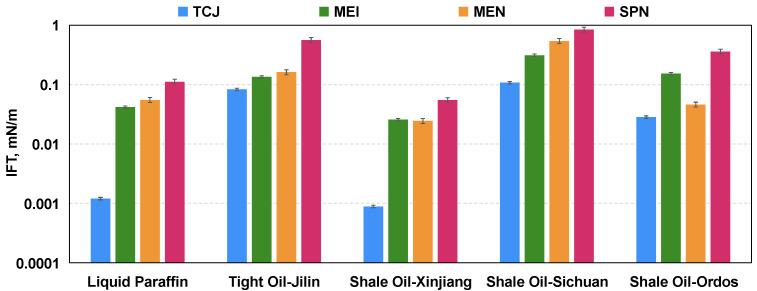
IFT reduction effect of four aqueous solutions (0.3 wt%) with oil from typical shale and tight formations of China.

In the experiments, the IFTs of four types aqueous solutions with the liquid paraffin and the dead oil retrieved from Jilin, Xinjiang, Sichuan, and Ordos basins of China were measured. The concentration is 0.3 wt%, which is a common concentration used in the EOR operations of oilfields. As shown in Figure 5, all chemicals have a significant IFT reduction effect, reducing the IFT of the oil and water phases to below 1 mN/m. In all cases of different oil samples, the IFT of surfactant TCJ is the lowest. This agrees well with research reports [35,36,37]. Ultra-low IFT (<10^−3^ mN/m) was achieved by TCJ with one oil sample, which is beneficial for maximizing the oil-sweeping effect of chemical fluids. Micro-emulsion-based imbibition agent MEI and nanomaterial MEN could also reduce IFT to a low level. Compared with surfactant-based systems, the interfacial effect of SiO_2_-based nanoparticle SPN is not prominent.

**Figure 6:**
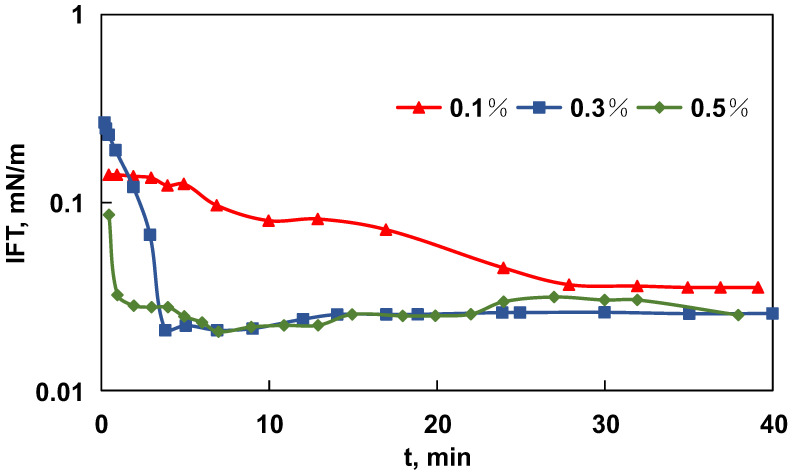
The IFT of the MEN with Xinjiang shale oil system at different concentrations against experiment time.

In addition, the IFT equilibrium curve of MEN at different concentrations (0.1 wt%, 0.3% wt%, and 0.5% wt) is plotted in Figure 6. When concentration increased from 0.1 wt% to 0.3 wt%, the IFT reduction effect became more significant. When concentration was raised to 0.5 wt%, there was no apparent change. According to previous researches, the CMC of those chemicals is typically in the range of 0.3–0.5 wt%. The IFT curves shows MEN achieved IFT equilibrium in a short period of time (<10 min), which is a profit, meaning that once the two phases come in contact, chemicals can quickly interact with crude oil.

The optimal IFT value for increasing Shale oil production is still under discussion. Some reports indicate that solutions with ultra-low IFTs exhibit better performance than those with relatively high IFTs [38,39]. Other reports suggests that pursuing ultra-low IFT is not necessary for unconventional EOR [40,41]. A few reports proposed that there is no monotonous relationship between IFT and imbibition rate [42,43]. A consensus is that there exists an IFT value range that enhances the imbibition and oil-sweeping effects, thereby improving oil recovery.

**Figure 7:**
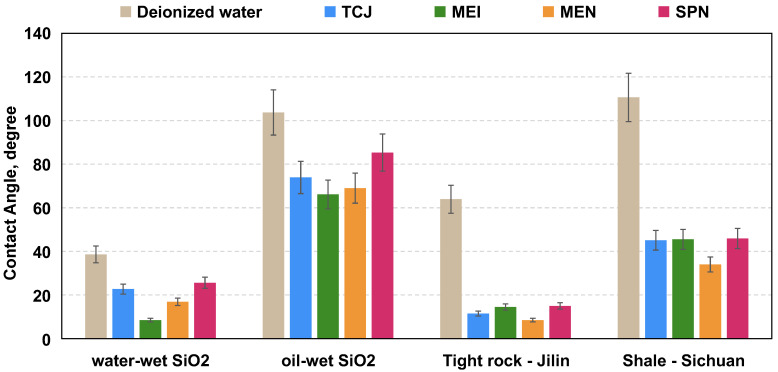
Wettability alteration effect of deionized water and aqueous solutions (0.3 wt%) on hydrophilic and hydrophobic surface.

The wettability of water-wet SiO_2_ surface, oil-wet SiO_2_ surface and oil-wet rock surfaces were altered by different chemical systems and the equilibrium contact angles were measured. The result is shown in Figure 7, which indicates that the aqueous solutions tested have a wettability alteration effect. The chemicals turned the oil-wet surface into water-wet, and altered the hydrophilic surface to strong water-wet. This phenomenon is named bi-phasic wettability property [44]. The microscopic wetting of shale surface is complicated. The chemicals can alter the surface to more water-wet state regardless of whether the initial wettability is oil-wet or not. This is the advantage of those chemicals producing significant effect. Among the evaluated chemicals, the MEI and MEN exhibited the most significant wettability alteration effect. Micro-emulsions have high oil film removal efficiency, as the oil phase in the emulsions could increase the solubility of the crude oil.

The wettability has a strong influence on the spontaneous imbibition rate. A few reports suggest that the wettability dominate the unconventional EOR process in the way of spontaneous imbibition, instead of IFT reduction [15,45]. As mentioned in the previous sections of this article, improving the recovery of shale and tight reservoirs is much more complex than the imbibition process, and the ability to alter wettability is only a part of the consideration when selecting chemicals.

#### 3.1.2. Hydrodynamic Radius and Micro-Morphology

**Figure 8:**
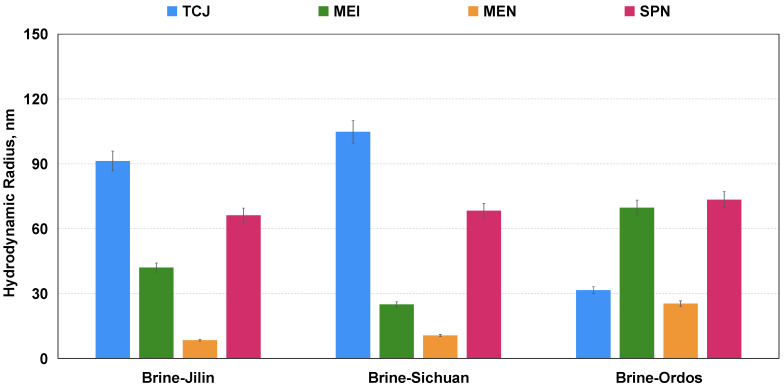
Hydrodynamic radii of four aqueous solutions measured in brine prepared according to the recipe of produced water from Jilin, Sichuan, Ordos Basins.

Hydrodynamic radii of the four aqueous solutions were measured in the brine prepared according to the recipe of formation produced water. The result in Figure 8 shows that salinity of the brine has an impact on the radius of the chemicals, especially the surfactant TCJ. Surfactant molecules form micelles and aggregates in the aqueous phase, thereby increasing the radius of the system. Nanoparticles also have the tendency to aggregates in order to lower the surface energy [46]. The size of MEN is the smallest, with an initial radius of approximately 10 nm. Its core–shell structure (oil phase as core, surfactant as shell), with hydrophilic groups arranged outward, avoids molecular aggregation.

**Figure 9:**
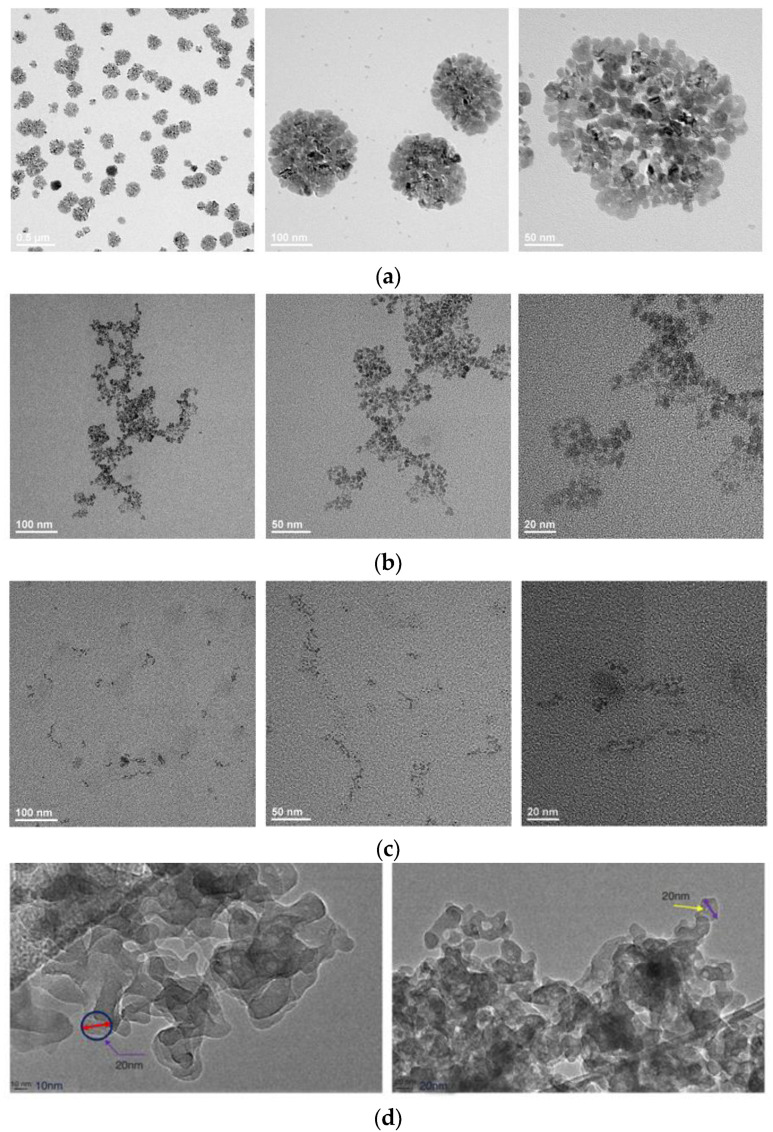
TEM observations of aqueous solutions. (**a**) TEM observation of TCJ at different magnifications; (**b**) TEM observation of MEI at different magnifications; (**c**) TEM observation of MEN at different magnifications; (**d**) TEM observation of SiO2 nanoparticles, reprinted with permission from Ref. [47] Copyright 2016 Elsevier.

The microscopic morphology of the chemical was observed using transmission electron microscopy, which can also verify the molecular size of the tested chemical. As shown in Figure 9, the micelles of surfactant TCJ were evident, and MEI also exhibited significant aggregation. MEN has the smallest size and was difficult to observe molecules using TEM. This is consistent with hydrodynamic radius measurement results. According to research [47], the aggregation of nanoparticles is very severe and can be easily observed using TEM or SEM.

The size effect of chemicals is crucial in unconventional EOR. Shale and tight oil are saturated in the nanopore and throat system of the formations. A small molecular size is beneficial for aqueous system to penetrate into the matrix through driving force or spontaneous absorption. That is why hydrodynamic radius measurement and micro-morphology observation are important steps in evaluating chemicals used in unconventional EOR. The core–shell structure of micro-emulsions helps avoid this problem and maintains its nanometer size under various conditions. This is the unique advantage of micro-emulsions or namely nano-emulsions in shale oil development.

#### 3.1.3. Adsorption and the Core Damage Rate

The adsorption loss of chemicals injected into the formation is a problem that hinders the wider application of chemical EOR. This study measured the adsorption rate of different chemicals on oil sand. The concentrations of the chemical was calculated from the surface tension measured initially and after rounds of adsorption. The results are summarized in Table 2. After the first round of adsorption, approximately half of the TCJ and SPN adsorbed on the solid surface were lost. After three rounds of adsorption, the loss rate of SPN exceeded 80%. This is due to its large surface area. The competitive adsorption between nanoparticles and oil on the surface of rocks is an important mechanism for nanoparticles displacing oil [48]. However, the adsorption loss of chemicals on the wellbore and fractures leads to low efficiency. The adsorption rate of MEN is the lowest, which is attributed to its core–shell structure. Presumably, a lower adsorption rate means more opportunity for the chemical to contact with oil in the tight matrix.

Chemicals can cause formation damage and reduce the permeability of rock through interacting with clay minerals, blocking the flowing channels and nanopores. The core damage is tested based on the permeability recovery rate. Due to the ease of measurement and more accurate results, rock cores with a relatively high permeability were used. The results in Table 3 indicate that all four chemicals caused a reduction in core permeability. The permeability recovery rates were above 70%, indicating that the core damage of these chemicals is not severe. MEN had the highest recovery rate and the least damage to rocks.

### 3.2. Micro-Oil-Displacing Performance

#### 3.2.1. The Threshold Pressure Gradient

**Figure 10:**
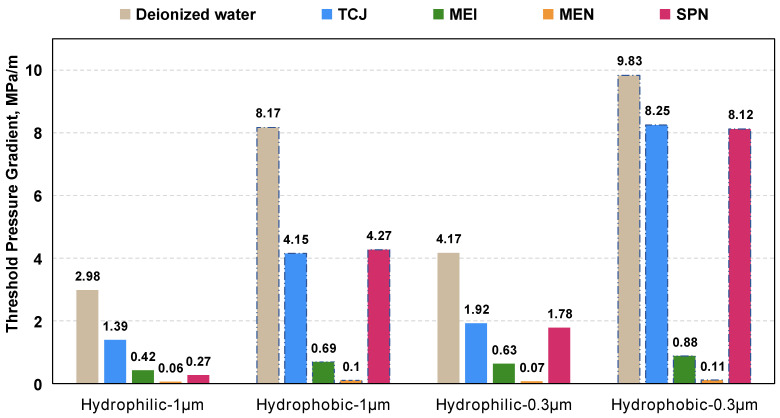
The threshold pressure gradient measured in hydrophilic and hydrophobic capillary tubes.

This study measured the threshold pressure of the chemical using hydrophilic and hydrophobic capillaries. The magnitude of the threshold pressure gradient is related to the difficulty of chemical fluids entering and flowing within the capillary system. This self-designed physical model simulate the permeation of fluid through the throat in the process fluid flow in capillaries. Figure 10 shows the results of TPG measured within hydrophilic and hydrophobic capillaries with an inner diameter of 1 μm and 0.3 μm. TPG of water in the hydrophilic capillary is significantly higher than that in hydrophobic capillary. It requires a stronger driven force for water to flow on oil-wet surface. TGP in the 1 μm capillary was higher than that in 0.3 μm capillary. As the size of the capillary decreases, the difficulty of fluid flowing into the capillary significantly increases.

The aqueous solutions reduced the TPG to a certain extent. Compared with hydrophobic capillaries, these chemicals have a more significant effect on reducing TPG in hydrophilic capillaries. The TPG reducing effect of MEN is most prominent. In the 1 μm hydrophilic capillary, the TPG decreased by 91%, and in the 0.3 μm hydrophilic capillary, the TPG decreased by 98.9%. This is consistent with the measurement results of hydrodynamic radius, demonstrating that aqueous chemical systems with smaller molecular size are more likely to enter nanoscale space. The surfactant TCJ and nanoparticle SPN exhibited good TPG reduction effect in the 1 μm hydrophilic capillary. As conditions shift towards hydrophobicity and smaller tube shape, size effects gradually dominated. The aggregation of surfactant and nanoparticles hinders fluid entry into nanospace, and cannot exert the IFT reduction and wettability alteration effect.

The flow of the aqueous phase in tight reservoirs is non-Darcy flow, and the flow regime affects the oil recovery factor [49]. TPG significant affects the fluid velocity and restricts the pressure propagation in low-permeability reservoirs [50]. As the TPG decreases, the propagation of pressure will be accelerated and the energy provided by chemical injection could reach more regions. The threshold pressure gradient is a parameter that bridges the microscopic space with the microscopic field.

#### 3.2.2. Micro-Fluidic Model

**Figure 11:**
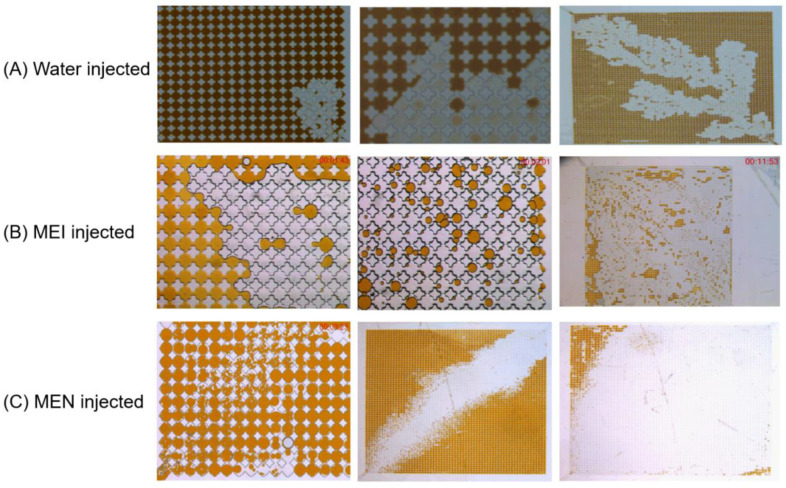
Photos captured during the displacing process of homogeneous micro-fluidic model with water, MEI, and MEN injected to displace oil.

Micro-fluid models are typically used to visualize multi-phase flow in porous media [51,52]. In this study, a homogeneous pore throat model was used to observe the micro-phenomena and behavior of the aqueous phases injected into an oil-saturated chip. Figure 11 shows the results of the chips saturated with shale oil retrieved from Xinjiang Oilfield being displaced by water, MEI and MEN, respectively. In the model injected with water, the boundary between water and oil was clear. Water-driven and -displaced oil mainly relied on the thrust at the inlet. The swept area was limited and the oil recovery rate was low. When injecting MEI, the oil phase released from the micro-emulsion interacted with the crude oil, shaping the oil into droplets. Through IFT reduction, wettability alteration, and oil solubilizing effect, the swept area and the oil recovery rate were significantly increased. In the captured video and photo of injecting MEN, it was observed that the crude oil was turned into small-size droplets. The oil phase in the micro-emulsion weakened the molecular interaction between crude oil components. In addition to low IFT and water-wet surface, this small-size oil effect increased the fluidity of oil, making it easier to drive the oil saturated in the model out. Sweeping efficiency and the recovery rate were higher than 90%.

**Figure 12:**
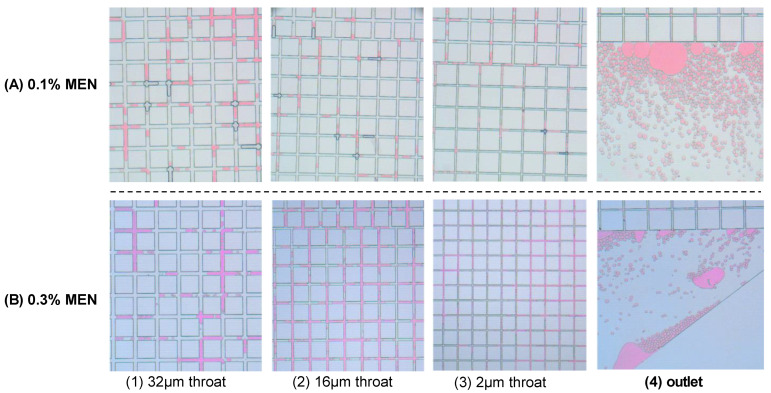
Photos captured during the displacing process of heterogeneous micro-fluidic model with (**A**) 0.1 wt% MEN and (**B**) 0.3 wt% MEN injected to displace oil.

In order to study the small-size oil effect in pores of different sizes, a heterogeneous model was designed and applied. The light shale oil was dyed with Sudan Red to increase the color contrast. Results in Figure 12 indicate that the size of oil droplets/oil flow can adaptively vary according to the size of the throat. As the radius of the throat decreased, the oil droplets became smaller. Small-size oil droplets can move quickly in the throat space. At the outlet, a large amounts of small oil droplets were observed. This small-size oil effect was significant when the concentration of injected MEN was 0.3 wt%. Similar behavior was observed injecting surfactant TCJ. Based on the results, we can speculate that when the pore size decreases to the nanoscale, MEN/TCJ can also change the size of the oil to the nanoscale. The aqueous solutions can enhance oil recovery by shaping oil into nanoscale droplets and increasing their fluidity. This is an important mechanism that contributes to incremental oil recovery from shale and tight formations.

#### 3.2.3. Core Injection and Fluid Penetration

**Figure 13:**
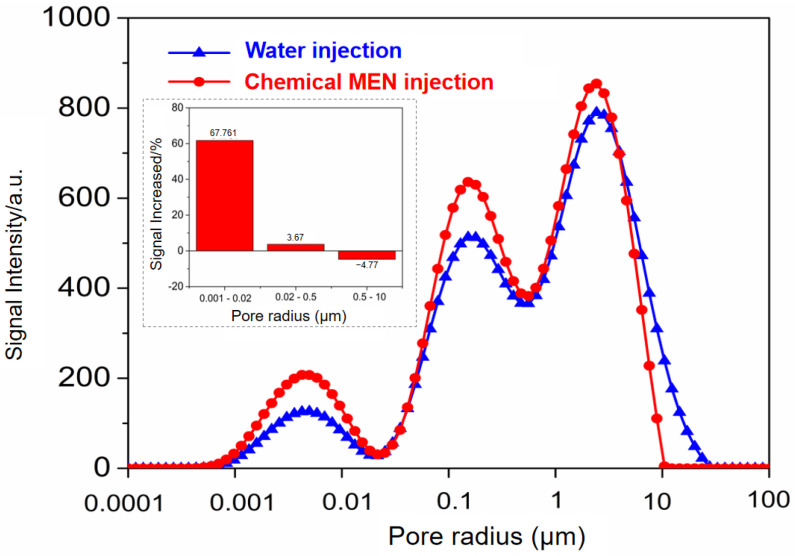
Plot of pore radius versus T2 signal intensity in the process of water injection and chemical injection. Change in signal percentage was calculated.

Fluid injection capability was evaluated using low-field NMR. Firstly, water was injected into the core and the distribution of the fluid in the porous medium is obtained through NMR analysis. Then, the chemical system MEN was injected into the core, and due to the different injection capabilities of water and chemical system, the distribution of fluids changed. As shown in Figure 13, the injected water inner the core was distributed in large pores (0.5–10 μm), medium pores (0.02–0.5 μm), and small pores (0.001–0.02 μm). After the MEN injection, the signal of fluid in medium and small pores increased significantly. The signal intensity in small pores increased by approximately 62%. The signal intensity in medium pores increased by approximately 4%. Although the peak intensity of medium pores increased, the range narrowed down. The signal intensity in large pores decreased, especially in pores of 5–20 μm.

The core injection experiment is a validation of the size effect of the aqueous solutions. The results indicate that compared to water, the aqueous phase containing chemical additives enhances the ability of fluid to penetrate into the nanopores. The inability to inject water into the matrix is the most important obstacle to the development of shale and tight formations in China. Especially after fracturing operations, the distribution of oil, gas, and water in the reservoir is very complicated. The ability to enter smaller pore spaces provides a foundation for chemical systems to play a subsequent role in reducing interfacial tension, improving wettability, and improving crude oil fluidity. Combined with NMR, this study verified that chemical systems that with small molecular size and good performance in the capillary and micro-fluidic model indeed have high injection capacity in nanoscale porous media.

Table 4 summarizes the performance of the solutions tested in this study. Three levels were used to describe their performance, I for good, II for moderate, and III for bad. It shows that these four solutions can reduce IFT and alter the wettability. Based on these two traditional tests, it is difficult to determine which one is the optimal choice. The droplet radius of the four solutions shows a significant difference. The droplet size of micro-emulsion MEN could be as small as 10 nm. Traditional surfactant and nano-fluid with solid particles have larger droplet sizes due to their clustering or aggregation. Consistently, the threshold pressure gradient of MEN is the lowest. It is easy for the nano-droplet to penetrate through channels or throats. A small-size oil effect was observed using the solutions. It was explained that the chemicals could break the association of heavy components in the crude oil and transform the oil from big clumps to small droplets. Fluid containing nanoparticles was not tested, thus it is unknown whether solid particles would block the instrument’s pipe. Finally, the overall performance of the solutions is given, and the results suggest that the micro-emulsion with the lowest droplet radius is the optimal selection for use as an agent in chemical EOR of tight and shale reservoirs.

### 3.3. Macro-Oil-Increasing Effect

#### 3.3.1. Pressure Field

**Figure 14:**
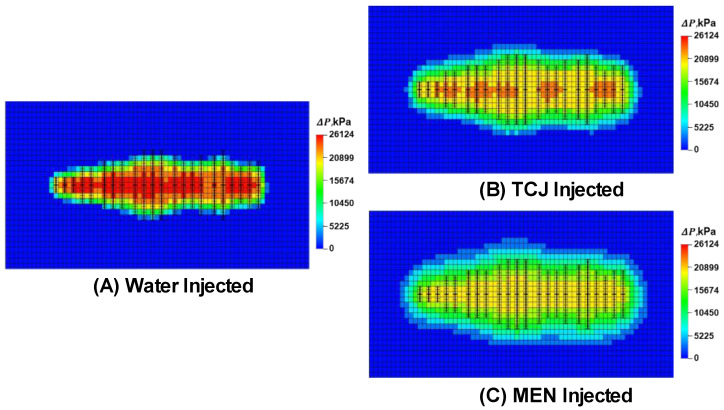
Numerical model of the fractured reservoirs with water, TCJ or MEN injected. The pressure field of the reservoir changed due to the fluid injection.

The numerical analysis of chemical effects on the reservoir/oilfield scale started from a fracture model established through determining the geological sweet spot and history matching the pumping pressure curves. On this basis, water or the aqueous chemical system was injected into the model. The threshold pressure gradient was used to describe the pressure propagation following fluid injection. The TPG of water injection, TCJ, and MEN measured in laboratory experiments was 4.17 MPa/m, 1.92 MPa/m, and 0.07 MPa/m, respectively. With this parameter added in the model, the pressure influence range of water, TCJ, and MEN was differentiated. The pressure of water injection in the area spread around the fractures. When TCJ was injected, the pressure change spread further. As shown in Figure 14, the micro-emulsion MEN has the lowest TPG, and pressure change can affect a large area. The pressure spread distance of water, TCJ and MEN injection was approximately 0.85~1.0 fold, 1.15~1.3 fold and 1.31~1.6 fold that of the hydraulic fracture length. According to grid calculation, the volume affected by water, TCJ and MEN injection was 117,400 m^3^, 178,360 m^3^, and 247,320 m^3^, respectively.

The pressure field is important in describing the effect of chemicals on shale and tight formations. In the fracture model, pressure difference is vital for the inflow of aqueous phase and the outflow of oil. In field practice, chemical fluid is pumped into the reservoir under high wellhead injection pressure, so that the fluid could enter the subsurface space as much as possible. By considering the size effect of chemicals and reducing the TPG, the range of pressure influence can be expanded and sweeping efficiency of fluid can be improved.

**Figure 15:**
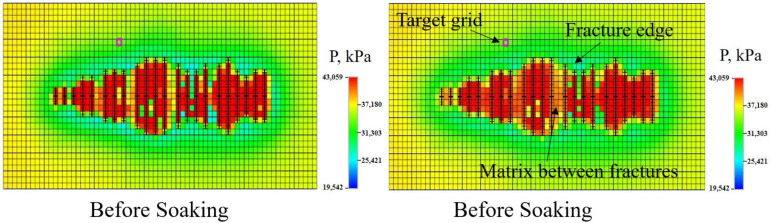
Model considering soaking after fracturing and fluid injection. The reservoir was divided into zones, matrix between fractures, fracture edge, and target grid that is away from fracture.

The effect of soaking well (shut-in) was also considered using the model. Soaking is a typical operation usually conducted by operators in the shale oil and gas industry. The benefits of soaking are still under discussion [53], and some researchers are skeptical about leaving fracturing water in the well to improve performance. This study simulated the effect of soaking on pressure field. During the period of soaking, high pressure caused by the fracturing reformation gradually propagated to the matrix between fractures and the fracture edges. As a result, the pressure at the matrix between the fractures increased by 2.5 MPa and the area near the fracture edges increased by 1.1 MPa. The target grid, shown in Figure 15, represents the zone that is far away from fractures. The pressure of the target grid decreased by 0.15 MPa. This indicates that soaking is beneficial for recovering the crude oil in the matrix near fracture/wellbore area, while resulting in pressure loss in areas far away from fractures. Therefore, the soaking time cannot be long; otherwise, the oil in the matrix away from fractures can merely be recovered.

#### 3.3.2. Oil-Increasing Mechanism

**Figure 16:**
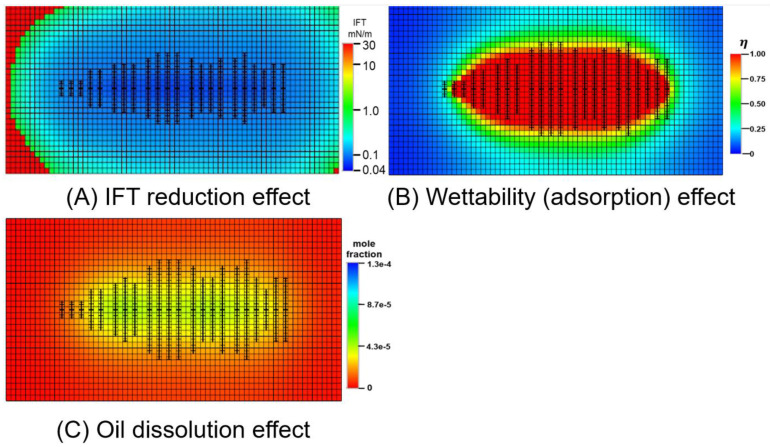
Model considering different oil-increasing mechanisms, including IFT reduction, wettability alteration, and oil dissolution.

The transformation effect on the reservoir of different oil-increasing mechanisms was studied using the numerical model. As previous researches reported, major mechanisms of chemical EOR are IFT reduction, wettability alteration, and oil dissolution. After injection of the micro-emulsion MEN, the distribution of IFT, the adsorption rate, and the mole fraction of oil in the aqueous phase are mapped in the fracture model. The results in Figure 16 show that the IFT of the largest area is below 0.1%. This is because IFT can be reduced to a low level with low concentrations of chemicals. The boundary of the IFT reduction effect can be recognized as the front of the micro-emulsion spread area. The wettability effect is defined by the adsorption rate. Only in the area close to the fractures, the adsorption rate exceeded the critical adsorption where oil-wetting is altered to water-wetting. In the zone away from the fractures, adsorption of chemicals is lower and wettability of the area is still oil-wet. Oil dissolution by micro-emulsion happens in the matrix between the fractures. Based on the distance of influence zone, this study suggests that the micro-emulsion increases oil by dissolving the oil in the matrix between the fractures, altering wettability in the matrix around the fractures, and reducing IFT in the whole area where fluids spread.

#### 3.3.3. Incremental Oil Production

**Figure 17:**
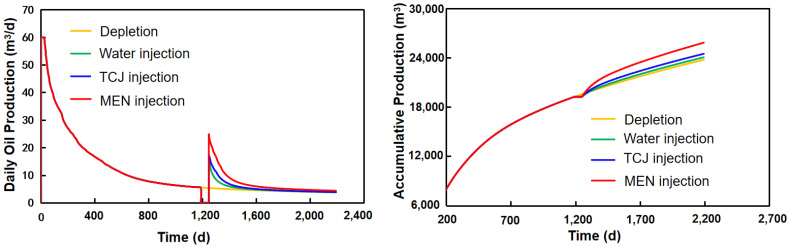
Oil production prediction with water, TCJ, and MEN injected into shale formation.

Oil production that can be increased by injection of chemicals and a huff-n-puff operation was predicted by the numerical model. The modeling results are shown in Figure 17. The oil well had been producing by depletion for 1200 days before fluid injection. After the fluid was injected, a time period of soaking was carried out. Daily oil production increased significantly after injection and soaking. During the huff-n-puff stage, the average daily oil production of water, TCJ, and MEN injection is 6.59 m^3^, 7.73 m^3^, and 10.54 m^3^, respectively. Compared to depleted production, injection of water, TCJ, and MEN increased the daily oil production by 49.43%, 75.28%, and 139%. Accumulated oil production was increased by 1.26%, 3.06%, and 8.74%, respectively.

The model prediction shows that the injected chemicals have an oil incremental effect and have good application prospects in the field. However, there is still a gap between numerical calculations and actual situations. The oil-enhancing effect of these chemicals still needs to be verified through extensive field tests. This study is an exploration in using a fracture model to discuss the effect of chemical EOR in shale and tight reservoirs. How to improve the fitting degree between numerical models and the field and improve prediction accuracy will be the focus of future research.

## 4. Conclusions

In this comparison study, a systematic process was established to evaluate the performance of chemicals in enhancing oil recovery in shale and tight reservoirs. Four typical aqueous solutions including betaine surfactant, a micro-emulsion-based imbibition enhancement agent, a micro-emulsion-based nanomaterial, and a solid particle-based nanomaterial were tested and their characteristics were compared. After three parts of experimental and numerical analysis, this study has drawn four main conclusions and some insights:

(1) Consistent with the traditional understandings, solutions have the ability to reduce oil–water IFT to a certain level and alter wettability to water-wet whether it is a traditional surfactant or in the form of micro-emulsion and nano-fluids. Moreover, the appropriate adsorption rate and low core damage are the indispensable requirements for chemicals applied in EOR.
(2) Unlike conventional reservoirs, water flooding does not work in tight reservoirs. Considering the size effect caused by nanoscale pores and throats in shale and tight reservoirs, this study suggests that small molecule sizes should be given priority when evaluating and selecting chemical systems.
(3) The comparison analysis results demonstrate that the micro/nano-emulsion with the lowest hydrodynamic radius is the optimal selection. This is attributed to the properties of the nano-emulsion matching well with the physical properties of shale and tight reservoirs.
(4) The aggregation behavior of the surfactant molecules and nanoparticles increases the apparent size of the aqueous system. Although both have their own advantages, it is still necessary to solve the problem of aggregation in order to effectively apply them in unconventional EOR.

Some insights were also gleaned from the comparison analysis:

(1) A small-size oil effect of the chemicals was observed in the microfluidic model. Due to the injection of chemicals, the shape of crude oil changed from a continuous aggregate to small droplets. The heterogeneous model with different sizes of throats indicates that the size of droplets can adapt to the size of the throat. As the throat narrows, the size of the oil droplets also decreases. This small-size oil effect of chemical additives should be given more importance in projects recovering shale and tight oil.
(2) The pressure field of the formation is transformed by injection of the aqueous solutions. Compared with water injection, the pressure propagation range of surfactant and micro-emulsion injection is wider due to reduced TPG. Oil production can be increased by different mechanisms, and the efficiency of those mechanisms has certain zones. Prediction of daily and accumulative oil production showed the application prospects of chemical EOR. Numerical modeling points out the direction for future research focus as well.

## Figures and Tables

**Table 1:** Solutions used in the EOR researches of typical shale and tight formations.

Solutions	Characteristic	Target Basin	Conclusions Drawn	Source
Surfactants (anionic and non-ionic)	Wettability alteration and IFT reduction on the spontaneous imbibition process	Sichuan, China	The anionic surfactants produced oil more than the non-ionic surfactants because it changed oil-wet shale to more water-wet.	Liu et al., 2019 [15]
Surfactants (fluoride and gemini)	Fluoride changed the wettability to oil-wet, while the gemini surfactant has strong interfacial activity	Ordos, China	Reducing the oil–water IFT and altering the wettability can achieve pressure reduction and increase fluid injection. Fluorine had more obvious pressure reduction.	Liu et al., 2022 [16]
Micro-emulsion (Nano-emulsion)	Changing wettability and reducing IFT to 0.0038 mN/m at 0.2 wt%,	ShengLi Oilfield, China	In addition to IFT reduction and wettability alteration, the emulsification and solubilization effects are revealed to be the dominant mechanisms.	Qu et al., 2022 [17]
Nano-fluids (consisting of nanoparticles)	Average hydrodynamic diameter was 2.8 ± 0.4 nm	Bakken, United States	Both the disjoining pressure and synergistic effect between the surfactant and nanoparticles benefit oil recovery.	Zhou et al., 2020 [18]
micro-emulsion-based nano-fluids	Ultra-low IFT between oil and water, bring down the IFT to the value of 0.001 mN/m	Not Given	The implementation of nanoparticles nano-fluid stabilizes the surface-active materials. It requires lesser quantity of surfactant.	Mariyate et al., 2021 [19]

**Table 2:** Adsorption calculated from surface tension and corresponding concentration.

Chemical	Surface Tension (mN/m)				Concentration (%)				Absorption Loss Rate (%)
	Initial	First Round	Second Round	Third Round	Initial	First Round	Second Round	Third Round	
TCJ	30.730	31.462	32.068	32.957	0.2	0.124	0.094	0.063	62.59
MEI	39.164	39.326	40.915	42.843	0.3	0.261	0.141	0.067	77.78
MEN	32.093	35.946	36.958	37.389	0.6	0.552	0.343	0.280	53.36
SPN	25.443	27.548	28.129	29.876	0.3	0.145	0.114	0.054	82.0

**Table 3:** Core damage tested from the permeability recovery rate after fluid injection.

Core	Chemical	Length (cm)	Diameter (cm)	Injection Pressure (MPa)	Initial Permeability (mD)	Permeability after Injection (mD)	Permeability Recovery Rate (%)
1#	TCJ	10.015	2.499	0.260	26.4530	14.2010	53.68
4#	MEI	10.106	2.506	0.218	31.7302	11.0875	34.94
2#	MEN	10.024	2.503	0.232	29.9381	17.5948	58.77
3#	SPN	10.094	2.492	0.214	32.4305	13.2333	40.81

**Table 4:** Comparison of the solutions in tabular form.

Solution	Type	IFT Reduction	Wet	Droplet Radius	Cluster	Adsorption	Core Damage Rate	Threshold Pressure Gradient	“Small-Size Oil” Effect	General
TCJ	Traditional surfactant	I	II	III	III	I	I	II	II	II
MEI	Micro-emulsion	II	I	II	II	II	III	II	II	II
MEN	Micro-emulsion	II	II	I	I	I	I	I	I	I
SPN	Nano-fluid with nano particles	II	II	II	III	III	II	III	Not tested	III

## Data Availability

Data are contained within the article.
